# The promotion of healthy breakfast and snacks based on the social marketing model: a mixed-methods study

**DOI:** 10.1186/s41043-021-00245-y

**Published:** 2021-05-07

**Authors:** Firoozeh Mostafavi, Fereshteh Zamani-Alavijeh, Marjan Mansourian, Fatemeh Bastami

**Affiliations:** 1 Department of Health Education and Promotion, School of Health, Isfahan University of Medical Sciences, Isfahan, Iran; 2 Department of Epidemiology and Biostatistic, School of Health, Isfahan University of Medical Sciences, Isfahan, Iran; 3 Department of Public Health, School of Health and Nutrition, Lorestan University of Medical Sciences, Khorramabad, Iran

**Keywords:** Breakfast, Snack, Social Marketing Model, Student, Mixed-methods study

## Abstract

**Background:**

Skipping breakfast and replacing it with non-nutritious snacks are progressively increasing among adolescents. This study aimed to develop an educational intervention based on the Social Marketing Model and evaluate its effects on healthy breakfast and snack consumption among female adolescent students.

**Methods:**

This mixed-methods study was conducted in 2016–2019 in two phases. In the first phase, a qualitative study was conducted through directed content analysis in guidance schools in Khorramabad, Isfahan, and Tehran, Iran, to explore factors affecting breakfast consumption. The results of this phase were set in the benchmarks of the Social Marketing Model. In the second phase, a randomized controlled trial was conducted based on the benchmarks of the Social Marketing Model on 94 students randomly recruited from guidance schools in Khorramabad, Iran.

**Results:**

The findings of the qualitative phase were categorized into the benchmarks of the Social Marketing Model, namely the social marketing mix, the intended behavior, internal and external competing factors for behavior modification, theoretical concepts related to the behavior, and the role of supporters. In the quantitative phase, the univariate analysis showed significant between-group differences concerning the product, price, promotion, and behavior (*p* < 0.05).

**Conclusion:**

Healthy breakfast and snack consumption can be promoted through making acceptable the tastes, costs, preparations, and consumption places of breakfast and snack.

**Trial registration:**

The trial was registered in the Iranian Registry of Clinical Trials (code: IRCT20170201032347N1).

The trial was registered in 11/07/2018 and is accessible on the Iranian Clinical Trial Registration website.

**Supplementary Information:**

The online version contains supplementary material available at 10.1186/s41043-021-00245-y.

## Background

Breakfast is a key component of healthy eating. It contributes to normal growth and development, particularly among children and adolescents. Yet, breakfast skipping is common among children, adolescents, and women [1]. Its prevalence in the USA and Europe is 10–30% [2–4]. Moreover, the prevalence of irregular breakfast consumption among Iranian children is 8–30% [5–7]. Breakfast consumption can significantly affect health. School-age children who consume breakfast report better eating profile than their peers who do not consume breakfast. Breakfast consumption improves cognitive and social functioning as well as mood [8–10], while its skipping is associated with limited intake of essential nutrients, inadequate physical development, impaired cognitive functioning, and behavioral problems among children. Moreover, breakfast skipping and its subsequent increased rate of unhealthy snack consumption can increase the risks of obesity and chronic health conditions and may result in academic failure [11, 12].

Because of the high prevalence of breakfast skipping and its association with different health problems, client-centered health education programs which consider ecological factors are recommended to promote healthy breakfast and snack consumption. The Social Marketing (SM) Model is one of the client-centered models for the modification of eating behaviors [13–15]. In SM, the principles and the techniques of commercial marketing are used to address the needs of the intended clients and to promote a given optimum social behavior among them through reducing its barriers and modifying its contributing factors [16].

Many studies have been conducted so far to identify and modify factors affecting breakfast and snack consumption behaviors [17–19]. Although they provided valuable information about these factors, they failed to identify the factors which market these behaviors. Moreover, SM-based studies in this area mainly dealt with the effectiveness of SM in promoting eating and food selection behaviors, but provided no comprehensive information about factors affecting these behaviors [20, 21]. In addition, most previous studies in this area were not client-centered, did not address competing factors affecting breakfast and snack consumption behaviors, did not use theoretical concepts to understand these behaviors and develop behavior-modifying interventions, and did not use the components of the SM mix (namely product, price, place, and promotion) for marketing these behaviors [22].

Promotion of healthy breakfast and snack consumption necessitates campaigns based on SM benchmarks [23]. These benchmarks should be modified and adapted according to the immediate context. A former study in Iran used the SM mix to explore healthy breakfast consumption [8]. However, the other social SM benchmarks (including the intended behavior, internal and external competing factors for behavior modification, theoretical concepts related to the behavior, and the role of supporters) in this area have not yet been explored in the sociocultural context of Iran [24]. Thus, the present study was conducted to address this gap. The aim of the study was to develop an educational intervention based on SM and evaluate its effects on healthy breakfast and snack consumption among female adolescent students.

### Objectives

This mixed method study was conducted in two main phases. The first phase was a qualitative study to explore participants’ personal experiences of the factors affecting healthy breakfast consumption, while the second phase was a quantitative study to evaluate the effects of an educational intervention developed based on the results of the first phase on healthy breakfast and snack consumption.

## Methods

### Iranian educational system

In Iran, schools are divided to two categories such as governmental and private ones. They have consistency of their programs, low authority and low flexibility in the method, content, and calendar of education. Iranian educational system includes prominent features such as variety in titles, registration conditions, tuition fees, and management procedures in school. In the present study, sampling was done from both types of schools in qualitative as well as quantitative phases. By doing so, the criterion of maximum variation has been observed and people from all socio-economic classes of have been included in the study**.**

### Phase I: the qualitative study

#### Design and setting

This descriptive qualitative study was conducted in 2016–2018 in two boys’ and four girls’ guidance schools in Isfahan, Khorramabad, and Tehran, Iran.

#### Participants

Study participants were 52 male and female students (with an age mean of fourteen), their parents, and school staff. All participants were Persian-speaker and Muslim and were purposively recruited from different socioeconomic status. In order to ensure maximum variation, participants from both governmental and private schools were selected in three different provinces, including Tehran, Lorestan, and Isfahan. The participant also included all socio-economic areas of the city and had diversity in terms of job and education of parents. Eligibility criterion was interest in sharing experiences of regular or irregular breakfast consumption, while exclusion criteria were unwillingness to stay in the study and inability to clearly explain experiences. Sample size was determined based on data saturation.

#### Data collection

Study data were collected through two focus group discussions with students, two focus group discussions with parents and school staff, and 52 in-depth semi-structured interviews. Each focus group discussion consisted of 5–7 participants and lasted 45–60 min. Interviews with participants in Isfahan and Khorramabad were conducted face to face, while interviews with participants in Tehran were conducted over the telephone. Interviews lasted 20–60 min. Each participant was interviewed just once. Broad open-ended questions were asked to start interviews and focus group discussions. Examples of these questions were “May you please explain about your experiences of the factors which contribute to healthy breakfast consumption?” “May you please explain about your experiences of the factors which contribute to morning anorexia?” and “How do you deal with tempting opportunities for consuming an unhealthy breakfast?” Subsequently, different probing questions were employed to more specifically focus on the study aim. All interviews and focus group discussions were tape-recorded and transcribed verbatim.

#### Data analysis

The data collected through interviews and focus group discussions were analyzed using directed content analysis based on the SM benchmarks. Two of the authors independently read and coded the transcripts and grouped the generated codes into SM benchmarks according to their similarities.

#### Rigor

Prolonged engagement in the study and spending adequate time for data collection helped us establish trust and rapport with participants and acquire detailed data. Moreover, sampling was performed with maximum variation to recruit participants with different characteristics from three large cities in Iran. This technique helped ensure the transferability of the findings. In addition, member checking was performed with several participants to ensure the consistency between their experiences and the findings of our data analysis [25].

### Phase II: the quantitative study

#### Design and setting

As a randomized controlled trial based on the SM benchmarks, the quantitative study was conducted from 2018 to 2019, in girls’ guidance schools in Khoraambad, Iran. The trial was registered in the Iranian Registry of Clinical Trials (code: IRCT20170201032347N1).

#### Sample

**Fig. 1:**
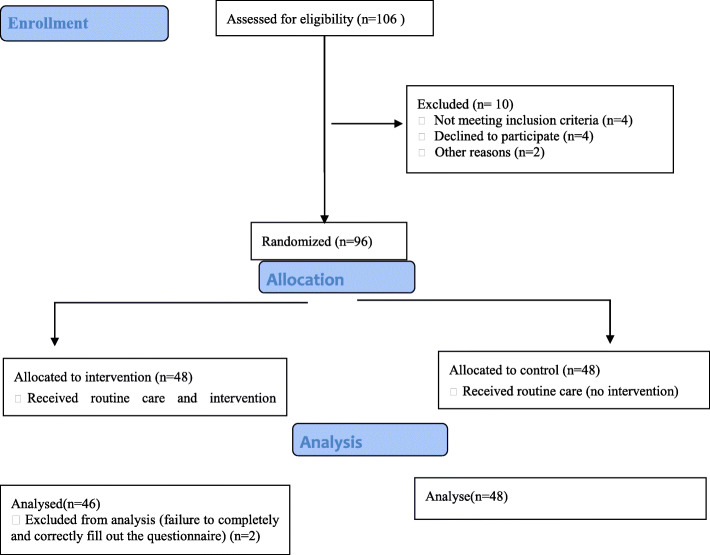
Consolidated Standards of Reporting Trials (CONSORT) flow chart for trial

Participants were female guidance school students. Eligibility criterion was agreement for participation and exclusion criteria were incomplete answering to study instruments and more than one absence from the intervention sessions. Participants were selected through multistage random sampling. Initially, Khorramabad city was divided into three hypothetical areas according to the socioeconomic status of its residents. The schools were selected randomly in every three socio-economic regions (two schools from each regions) and randomly located for intervention and control groups. The selected six schools were randomly allocated to a control group (three schools) and an intervention group (three schools). After that, a random sample of students was selected from each educational level (i.e., levels seventh, eighth, and ninth). Flow chart for trial recruitment is attached (Fig. 1).

Using the sample size calculation for the comparison of two means and with an attrition rate of 10%, sample size was calculated to be 48. Sample size calculation formula was, $n=\frac{{\left({Z}_{1-\alpha /2}+{Z}_{1-\beta}\right)}^2\ {S}^2}{d^2}$.

With a sample size of 48 per group, the study was powered to detect a large group difference on the primary outcome measure of *d*=0.8 with 80% power and a 2-sided a of 0.05 while accounting for a potential loss of power due to up to 10% of students lost to follow-up.

There are some representative variables in every three socio-economic regions (for example, educational level, occupation, income). Two intervention and control groups were compared according to these socio-economic variables, and the significant variables were adjusted in next modeling. Indeed, instead of multilevel analysis, we have controlled the differences between regions by adjusted analysis in modeling considering representative variables for different regions.

#### Intervention

Based on the SM campaign launched by the Colorado Nutrition Network for healthy eating promotion among the preschool children of low-income families [14], a ten-week healthy eating campaign was developed and conducted using SM strategies (Table 1). The theme of the program was “Test delicious homemade snacks”. The campaign was conducted at school level and all students and school staff were informed about the campaign through a healthy eating exhibition and campaign posters. Educations about healthy eating were integrated into the weekly educational programs of the students, and their teachers were asked to provide students, on a weekly basis, with educations about the consumption of healthy breakfast and snack. Moreover, the buffet staff of the schools were asked to replace potato chips and cheese puffs in the buffet shelves with healthy snacks (Appendix 1). Besides, a 10-week educational program on healthy breakfast and snack consumption was implemented for students. Initially, students in each school were divided into three small groups with 5–6 students from each educational level. Then, a 1-h educational session was held for each group. In the first session, healthy and unhealthy snacks were introduced to students through animations and posters, and then, animations were provided to students for personal use and posters were hanged on school boards. In sessions 2–9, each student made a healthy snack in the school kitchen in the presence of all her group members and provided explanations about the snack ingredients and its advantages. She had already learned how to make the snack at home from her parents. All her group members tested her snack. In the tenth session, all students made healthy homemade snacks and presented them in a healthy eating exhibition at school.

**Table 1:** SM mix adapted for the healthy breakfast and snack consumption

SM mix	Strategies
Product: The product strategy refers to the preferences which restrict the consumption of healthy breakfast.	Serving method (a homemade decorated snack served in a dish at school) Diversity (diversity in different meals instead of diversity in a single meal) Characteristics which show that the product is healthy (packed with the ingredient table on the package, to be homemade) The appearance of the package Taste Warm food serving
Price: The cost strategy refers to the readiness for paying the time-related, psychological, financial, and social costs of healthy breakfast consumption.	Reducing price/barriers through • Making snacks at home • Promoting perceived benefits and making prices acceptable through the theoretical concepts related to the behavior (i.e. fear over the complications of breakfast skipping, perceived self-efficacy, and perceived benefits • Implementing the intervention at school • Direct experience of snack making and preparation
Place: The place strategy denotes the pleasant physical and social environment for healthy breakfast consumption.	Healthy snack time at school Placing healthy snack in school buffet
Promotion: This strategy consists of the channels which promote the consumption of healthy breakfast.	Making the product, place, and prices attractive through formal and informal channels Educational methods and devices (animation, workshop, poster, painting, etc.)

Educational materials for the sessions were developed based on the theoretical concepts related to healthy breakfast and snack consumption identified in the qualitative phase of the study. These concepts were perceived self-efficacy, fear over the complications of breakfast skipping, and perceived benefits. Accordingly, based on the perceived self-efficacy concept, students were asked to explain about the preparation of an innovative healthy snack and show its preparation in a step-by-step approach. Moreover, they verbally and practically encouraged each other for the consumption of healthy breakfast and snack. Based on the perceived benefits concept, students were asked to talk with each other about the benefits of healthy homemade snacks such as their good taste, low cost, easy preparation and eating, and their positive physical, intellectual (or learning-related), emotional, and social effects. The role-paying method was used to deliver educations about these benefits. In addition, based on the concept of fear over complications, participants were provided with educations about the complications of breakfast skipping and unhealthy snack consumption such as weight gain, dental caries, and reduced immunity. Participants were also encouraged to discuss about the necessity of healthy breakfast consumption by both obese and thin people. Reinforcing educational messages were sent to parents and school staff through social networks. Virtual groups in social networks were also created for parents and school staff, where they were asked to share their experiences. Participants in the control group just received healthy nutrition services routinely provided at schools in Iran.

**Fig. 2:**
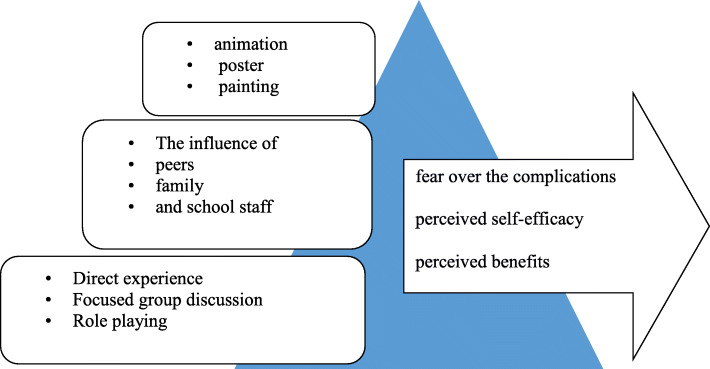
The implementation of the intervention based on the Edgar Dale’s Pyramid of Learning (cone of experience)

Educational intervention was carried out based on the Edgar Dale’s Pyramid of Learning [26] (Fig. 2). Three months after the end of the educational intervention, both intervention and control groups completed the post-test questionnaires.

#### Outcome measures

The primary outcome of the study was the factors affecting healthy breakfast consumption which were measured using the 38-item Factors behind Breakfast Consumption Scale. This scale contains four domains, namely product or preferences which can restrict healthy breakfast consumption, readiness to pay for costs, place-related preferences, and channels which promote healthy breakfast consumption. The items of the product domain were scored on a five-point scale from 1 (“Completely disagree”) to 5 (“Completely agree”). The items of the promotion domain were scored dichotomously as either 1 (“Yes”) or 0 (“No”). The items of the place domain were described using frequency distribution. The face, content, and construct validity of this scale were assessed and confirmed in a former study. That study reported that the four-domain structure of the scale explained 61.73% of the variance of the factors affecting breakfast consumption and the Cronbach’s alpha of the scale was 0.71 [27]. This scale was administered to participants before and 6 months after the onset of the study intervention.

The secondary outcome of the study was the healthy breakfast and snack consumption behaviors. These behaviors were assessed over a 7-day period using a scale with items scored 1–4.

#### Data analysis

Statistical data analysis was done using the SPSS software (v. 20.0) and at a significance level of less than 0.05. Categorical variables were described via absolute and relative frequencies, and numerical variables were described via mean and standard deviation. Between-group comparisons concerning categorical and numerical variables were made using the chi-square and the independent-sample *t* tests, respectively. The effects of confounders were removed through univariate analysis in three models which were respectively adjusted for pretest readings (Model 1), pretest readings and mother’s occupation (Model 2), and pretest readings, mother’s occupation, and parents’ educational level (Model 3).

## Results

### Results of phase I: the qualitative study

The results of the study included 201 first-level codes, successive classification of which resulted in the emergence of 18 subcategories, which were divided into five main categories, and led to the discovery of SM benchmarks about healthy breakfast and snack consumption including SM mix, identifying the intended behavior, internal and external competing factors for behavior modification, theoretical concepts related to the behavior, and the role of supporters.

#### SM mix

The components of the SM mix were explored and presented in a former study [8] and were used in the quantitative phase of the present study to develop the study intervention (Table 1).

#### Identifying the intended behavior

Two behaviors were identified, i.e., breakfast consumption at home and snack consumption at school.

When I awake, I have no appetite for breakfast and hence, eat snack at school (a student).

I eat breakfast at home because it is easier and more comfortable. Moreover, I can eat at home whatever I want for breakfast (a student).

#### Internal and external competing factors for behavior modification

Barriers and competing factors in breakfast consumption were stress, entertainments, high-calorie dinner, immobility, loneliness, and school vacation. Both students and parents highlighted that stress, nighttime entertainments (watching television and using tablet), lonely eating, sleep inadequacy, high-calorie dinner late at night, and morning boredom negatively affect appetite for breakfast. On the other hand, morning exercise can cause physical and mental happiness and positively affect morning appetite.

At exam days, my daughter has high levels of stress and hence, has no appetite for breakfast (a mother). In the morning, I have stress to be late at school and to be absent (a student). I stay awake late at nights to watch my favorite serials. Therefore, I awake late in the morning and have neither time nor appetite for breakfast (a student). If children eat a high-calorie dinner late at night, they may feel full in the morning and will have no appetite for breakfast (a mother). In the morning, I’m bored and do not value eating or what to eat. But, a little exercise increases my appetite (a student). When a child sees three glasses of milk on the table, he/she understands that other family members are going to eat breakfast. This can increase his/her appetite (a mother). In summer, almost all school-age children awake late in the morning and don’t eat breakfast (a mother).

#### Theoretical concepts related to the behavior

Factors which might facilitate students’ engagement in the breakfast consumption behavior were fear over the complications of breakfast skipping, perceived self-efficacy, and perceived benefits. Fear over the complications of breakfast skipping consisted of the two subcategories of fear over obesity and fear over illness.

Fear greatly affects children, particularly the girls. For instance, if a teacher attributes the ailment of a student to breakfast skipping, other students will understand the importance of breakfast consumption (a father).

Perceived self-efficacy also included two subcategories, namely responsibility assignment and vicarious experience. Participants highlighted that assigning responsibilities to students provides them with the opportunity to personally experience a given behavior and attempt to successfully show it. Moreover, vicarious experiences with peers, family members, and school staff can contribute to healthy breakfast consumption.

My daughter is thirteen-year-old and can make breakfast for herself. Of course, she previously was also able to make breakfast and she made breakfast for her father and brother when I was not at home (a mother). They provided us with a food made of lentil. I didn’t like it at all. But, when I saw my friends and teachers were eating it, I tested it and found it good (a student).

The perceived benefits of breakfast consumption came into four subcategories, namely physical, intellectual (or learning-related), emotional, and social benefits.

My children said that those mothers of their friends who didn’t eat breakfast were inattentive to them (a mother). In the morning, children are sleepy and hence, their attention is not adequately concentrated. However, breakfast eliminates their sleepiness (a mother). Last year, one of my classmates told me about the bad smell of my mouth and refused sitting next to me. Since then, I eat breakfast (a student).

#### Segmentation; the role of supporters

Parents and school staff were identified as the supporters of the breakfast consumption behavior. The more attentive the mothers and the school staff are to breakfast consumption by students, the more likely the students will consume breakfast. “Healthy snack time” at school can also encourage healthy snack consumption among students.

Before breakfast, I give them orange juice. Or for instance, as they like to eat flavored milk, I mix milk with honey or banana. They drink it with appetite (a mother). School staff have given us an eating plan and have required us to provide our children with healthy snacks, according to the plan, to be eaten at the healthy eating time at school (a mother).

### Results of phase II: the quantitative study

The means of participants’ age in the intervention and the control groups were 13.71±0.91 and 13.75±1.2, respectively. No statistically significant differences were observed between the groups concerning participants’ and their parents’ characteristics as well as the place of breakfast consumption (*p* > 0.05), except for parents’ educational level and mother’s occupation (Table 2). The mean score of restrictive preferences (product), readiness for paying the costs (price), and promotion channels and behavior improved significantly after intervention in intervention group (Table 3). The results of univariate analysis illustrated significant between-group differences concerning product, price, promotion, and behavior (*p* < 0.05; Table 4).

**Table 2:** Between-group comparisons concerning participants’ and their parents’ demographic characteristics as well as the place of breakfast consumption

Group/characteristics		Intervention			Control			*p* value*
		*N*	%	Total	*N*	%	Total	
School type	Public	24	52.2	46	28	58.3	48	0.60
	Private	22	47.8		20	41.7		
Educational level	Seventh	15	32.6	46	18	37.5	48	0.90
	Eighth	15	32.6		15	31.3		
	Ninth	16	34.8		15	31.3		
Father occupation	Self-employed	18	41.9	43	23	47.9	48	0.40
	Employee	23	53.5		20	41.7		
	Retired	2	4.7		5	410.4		
Mother occupation	Housewife	32	69.6	46	45	93.8	48	0.002
	Employee	14	30.14		3	6.3		
Father educational level	Below diploma	15	34.9	43	9	18.8	48	0.01
	Diploma	5	11.6		18	37.5		
	University	23	53.5		21	43.8		
Mother educational level	Below diploma	13	28.3	46	12	25	48	< 0.001
	Diploma	11	23.9		32	66.7		
	University	22	47.8		4	8.3		
Income level	Low	7	15.2	46	2	4.2	48	0.08
	Moderate	19	41.3		29	60.4		
	High	20	43.5		17	35.4		
Family size	3	5	10.9	46	7	14.6	48	0.80
	4	22	47.8		20	41.7		
	5 or more	19	41.3		21	43.8		
Birth rank	First	20	43.5	46	25	52.1	48	0.60
	Second	12	26.1		13	27.1		
	Third or more	14	30.4		10	20.8		
Breakfast consumption place	Home	10	21.7	46	15	31.3	48	0.50
	School	34	73.9		32	66.7		
	Doesn’t matter	2	4.3		1	2.1		

*The results of the chi-square test

**Table 3:** The comparison of the mean scores of product, price, promotion, and behavior in the intervention and control groups before and after the intervention

Variables	Groups	Before intervention		After intervention		*p* value^b^	Mean difference± standard deviation	95% confidence interval for difference	
		Mean	Standard deviation	Mean	Standard deviation				
								Lower	Upper
Restrictive preferences (product)	Intervention control	74.78 75.77	11.2 12.13	79.13 76.05	11.3 7.82	0.02 0.80	−4.65±12.5 −0.26±13.88	−8.06 −4.43	−0.63 3.90
	*p* value^a^	0.60		0.13					
Readiness for paying the costs (price)	Intervention control	57.22 57.81	20.8 13.04	64.28 60.16	10.09 14.55	0.01 0.46	−7.05±18.66 −1.75±15.75	−12.60 −6.48	−1.51 2.3
	*p* value^a^	0.80		0.12					
Promotion channels	Intervention control	26.87 28.73	20.16 9.80	65.99 29.68	15.60 14.03	<0.001 0.75	−39.13±21.8 −0.9±19.29	−45.6 −6.75	−32.65 4.84
	*p* value^a^	0.67		<0.001					
Behavior	Intervention control	62.17 63.52	14.9 6.1	70.9 64.2	11.86 9.2	<0.001 0.75	−8.73±14.73 −0.58±12.23	−13.1 −4.26	−4.34 3.08
	*p* value^a^	0.50		0.003					

^a^Significant, independent sample *t* test, ^b^Significant, paired sample *t* test

**Table 4:** The results of the univariate analysis for the effects of the study intervention

Group/variable	Intervention (mean±SD)		Control (mean±SD)		Model 1	Model 2	Model 3
	Before	After	Before	After	*p* value	*p* value	*p* value
Restrictive preferences (product)	74.78±11.2	79.13±11.3	75.77±12.13	76.05±7.82	0.02	0.01	0.05
Readiness for paying the costs (price)	57.22±20.8	64.28±10.09	57.81±13.04	60.16±14.55	0.05	0.004	0.01
Promotion channels	26.87±20.16	65.99±15.60	28.73±9.80	29.68±14.03	<0.001	0.01	0.008
Behavior	62.17±14.9	70.9±11.86	63.52±6.1	64.2±9.2	0.001	0.001	0.04

Model No. 1: Raw model

Model No. 2: Modified for school type

Model No. 3: Adjusted for school type, age, educational background, parents’ occupation, parents’ education, income level, family members, and birth rank

## Discussion

This study aimed to develop an SM-based educational intervention and evaluate its effects on healthy breakfast and snack consumption among female adolescent students. Study findings indicated significant between-group differences concerning product, price, promotion, and behavior, confirming the effectiveness of the study intervention in significantly promoting healthy breakfast and snack consumption among participants. Similarly, a review study reported the positive effects of the interventions which included at least five benchmarks of SM [23]. The effectiveness of the study intervention is attributable to the use of SM benchmarks such as using behavior modification theories, understanding competing factors, dividing clients into subgroups (i.e., students, parents, and school staff), using a mix of interventions (such as lecture-based, exhibition holding, role playing), and using strategies developed based on the SM mix.

Study findings showed significant between-group difference concerning the posttest mean score of preferences which restricted healthy breakfast consumption. The higher scores of such preferences denote that individuals select and consume healthier breakfast. In order to encourage the consumers of a given product, the characteristics of the product should have great effects on them [28]. Accordingly, we used a range of restrictive preferences which were indicative of a pleasurable healthy food. Studies showed that pleasurable characteristics of foods (i.e., taste, cost, and easiness) are motives for food selection [21]. Except for innate preferences for sweet taste and refusal of bitter taste, individuals acquire and modify their food preferences according to their own personal experiences, particularly during childhood. Frequent exposure to new and healthy foods (for 8–15 times) in a positive environment (such as school) can enhance the acceptance of those foods by individuals [29]. Accordingly, the study intervention was implemented in ten weeks using the SM mix and with the theme of “Test delicious homemade snacks” in order to expose students to healthy foods for 8–10 times.

Study findings also indicated the effectiveness of the study intervention in significantly promoting participants’ readiness for paying the time-related, psychological, financial, and social costs of healthy breakfast and snack consumption. The major challenges of SM are to reduce price and barriers and to use tangible or intangible motives to enhance clients’ engagement in the intended behavior [30]. Pleasurability of healthy foods and personal experiences in preparing them are motives for their consumption [31]. These motives were integrated into the study intervention so that students experienced the healthy snacks they had personally prepared. This fact had significant effects on their attitudes towards the deliciousness of healthy snacks [32]. Implementing the intervention at school level as well as the promotion of healthy snacks through appropriate channels also promoted students’ engagement in healthy breakfast and snack consumption. Besides, we used behavior-related theoretical concepts in order to reduce the different costs of healthy breakfast and snack consumption and make these behaviors acceptable for participants. These concepts were fear over the complications of breakfast skipping, perceived self-efficacy, and perceived benefit. Similarly, in several earlier studies into the effectiveness of a physical activity campaign, adolescents were asked to identify and perform their favorable physical activities [33].

Study findings also showed that participants in both groups preferred healthy breakfast consumption at school and with their friends over breakfast consumption at home. Place, as a strategy of the SM model, can reduce the costs of engagement in a given behavior [34]. It refers to the social environments in which the pleasurability of healthy food consumption is learned and promoted [35]. An SM-related systematic review showed that the use of interesting and frequently used places such as schools was the most commonly used SM strategy for behavior modification [36]. Students learn from their parents and other social agents such as school staff to like and consume certain foods. Therefore, parents and school staff should be trained to expose students to the preparation and use of healthy foods [21]. Moreover, school staff can promote healthy breakfast and snack consumption among students through providing them with healthy food consumption facilities (such as health snack time) and favorable social environments [37]. The way of presenting healthy foods in school buffet can also affect their pleasurability for students [21]. In the present study, competing snacks such as potato chips and cheese puffs which had occupied much space in school buffet were replaced with healthy snacks. This strategy gives signals to students that healthy snacks are also pleasurable. Contrarily, confining healthy snacks to certain buffet places (such as the shelf of healthy snacks) or confining their consumption to certain environments (such as the room of healthy snacks) can reduce their pleasurability [38].

In line with the findings of previous studies, our findings revealed that the use of appropriate and reliable channels for the promotion of healthy breakfast consumption significantly increased participants’ engagement in healthy breakfast consumption [39]. A key point in using healthy behavior promotion channels is that the communication should create appropriate cognitive responses in clients. For instance, if the communication arouses negative thoughts or imaginations about the message sender, clients will not be persuaded to engage in the intended behavior [40]. One of the aims of SM-based interventions is to use user-preferred channels to make a given product, its price, and its place pleasurable to its clients [41]. Thus, we employed strategies to make healthy breakfast and snack consumption pleasurable for students. On the other hand, evidence shows that highlighting the nutrient profiles of food stuffs may cause presumptions about their poor tastes, while highlighting their pleasurability can persuade their clients into testing and using them [41]. Channels to promote healthy breakfast and snack consumption in the present study consisted of both formal channels, i.e., teachers and healthcare providers, and informal channels, i.e., parents and peers. Previous studies reported that as role models or subjective norm sources, these individuals are the most important health-promoting channels [42, 43]. The other promotion channels in the present study were healthy eating exhibitions, posters, and animations. These channels promote healthy eating in environments which are not occupied by competing foods and hence, are more likely to promote healthy eating [44].

The ultimate aim of the study intervention was to promote healthy breakfast and snack consumption behaviors among students. In fact, the product strategy of SM was used for behavior modification and promotion [45]. Behaviors can affect beliefs, feelings, and internalization of health-promoting behaviors [46]. Behavioral habits can also affect health-promoting behaviors such as healthy breakfast and snack consumption [47]. In line with the findings of several former studies, our findings indicated unhealthy behaviors such as staying awake late at night and immobility among adolescents. These behaviors are competing factors affecting health-related behaviors [48].

In the present study, although the subjects in both intervention and control groups were randomly enrolled in both governmental and private schools, a greater number of mothers with higher education and employment were seen in the intervention group. The results of demographic studies on children’s health show that the level of maternal education as a background indicator improves children’s health. The level of maternal education of is related to the socio-economic status of the family, which determines the health of the children. In addition, the mother’s level of education may cause positive changes in behavior among children [49]. In the present study, although the two groups of students were identical in terms of most of the contextual variables, but in any case, differences in some characteristics of students’ parents can be considered a study limit. It is suggested that matching be done in future studies.

Among the strengths of the study was the context-based exploration of SM benchmarks for healthy breakfast and snack consumption through a qualitative study. One of the limitations of the study was that we did not perform message framing and testing due to time limitation.

## Conclusion

This study concludes that the multi-component SM-based educational intervention is effective in significantly promoting healthy breakfast and snack consumption among female adolescent students. This intervention can be further developed and used for the promotion of healthy behaviors among adolescents.

## Supplementary Information

**Additional file 1.**

## Data Availability

The datasets used and/or analyzed during the current study are available from the corresponding author on a reasonable request.

## Abbreviations

SM: Social marketing
