# Supplementary figures and images for: The promotion of healthy breakfast and snacks based on the social marketing model: a mixed-methods study
Source: J Health Popul Nutr. 2021 May 7;40:22. doi: 10.1186/s41043-021-00245-y (PMC8106147; doi:10.1186/s41043-021-00245-y)

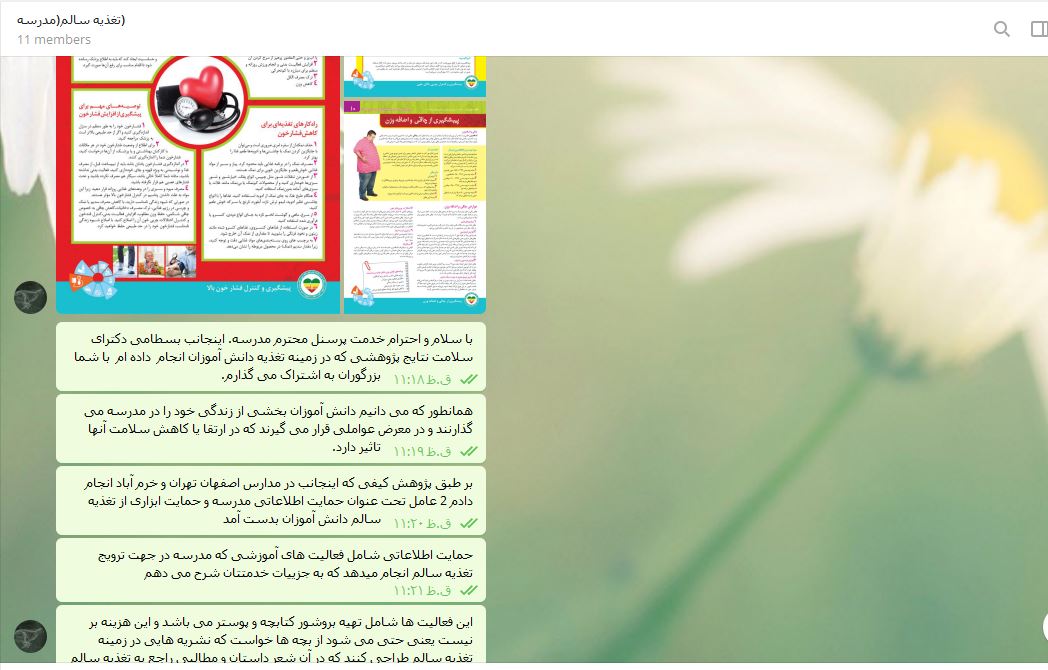


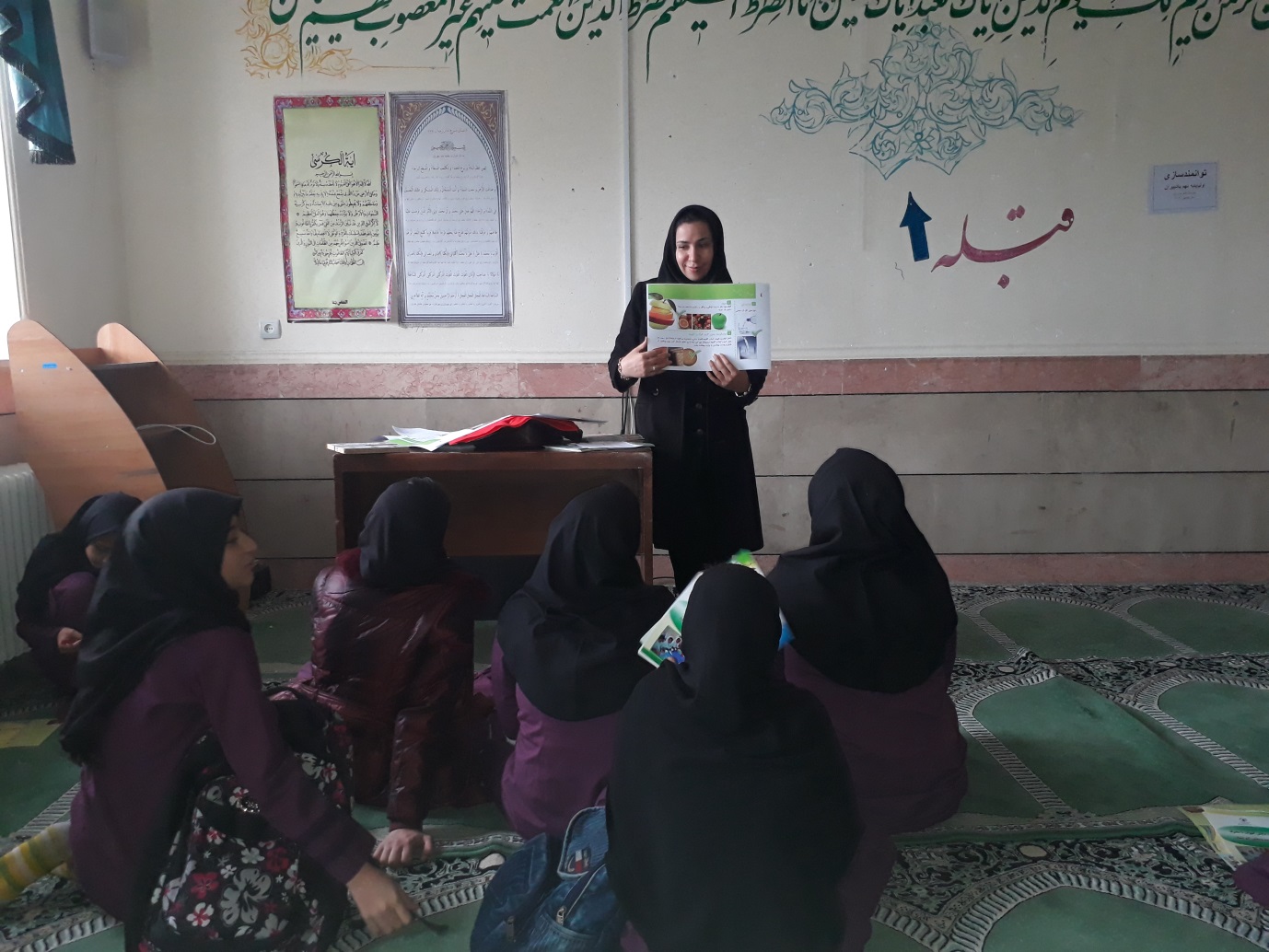


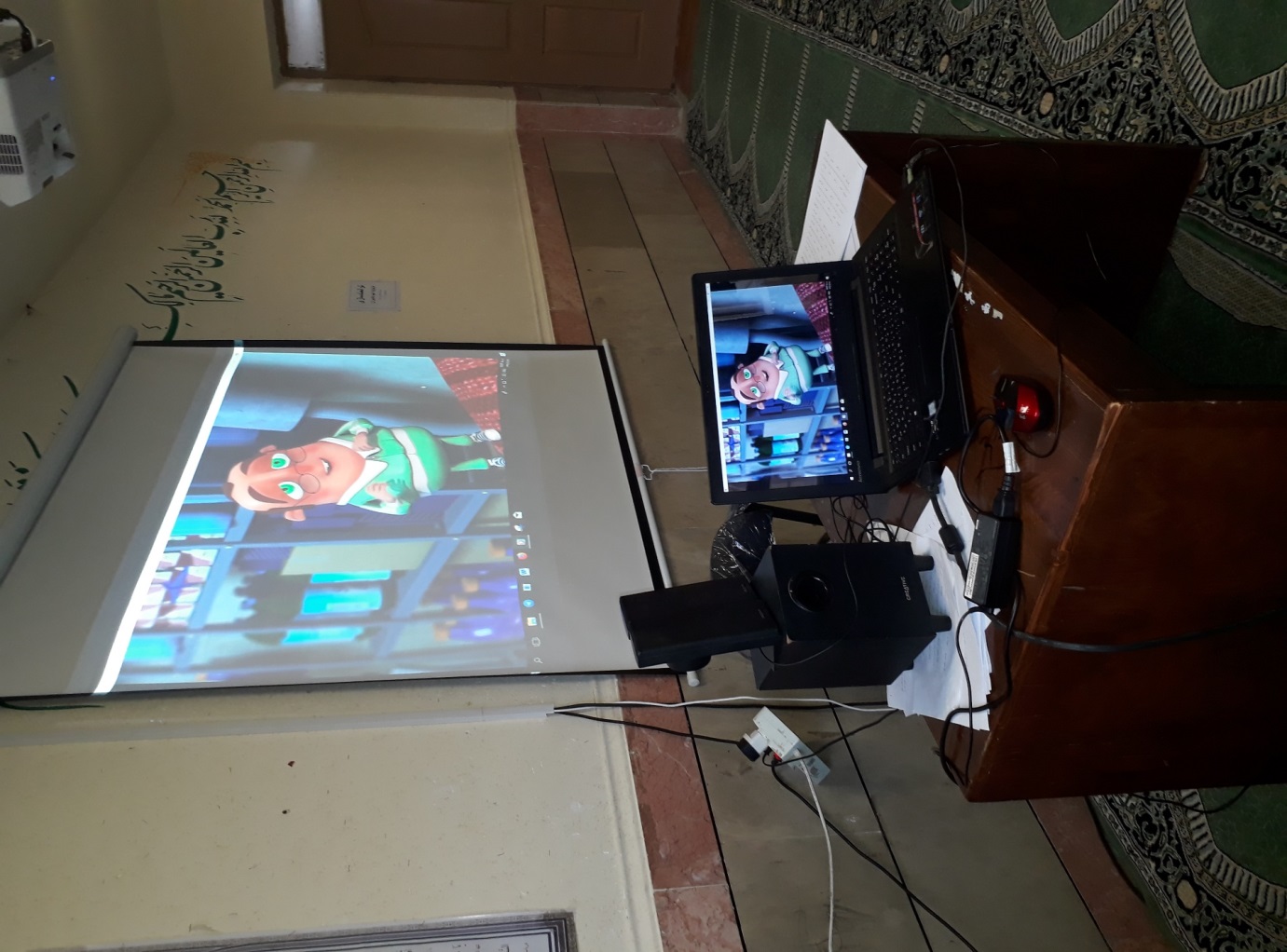


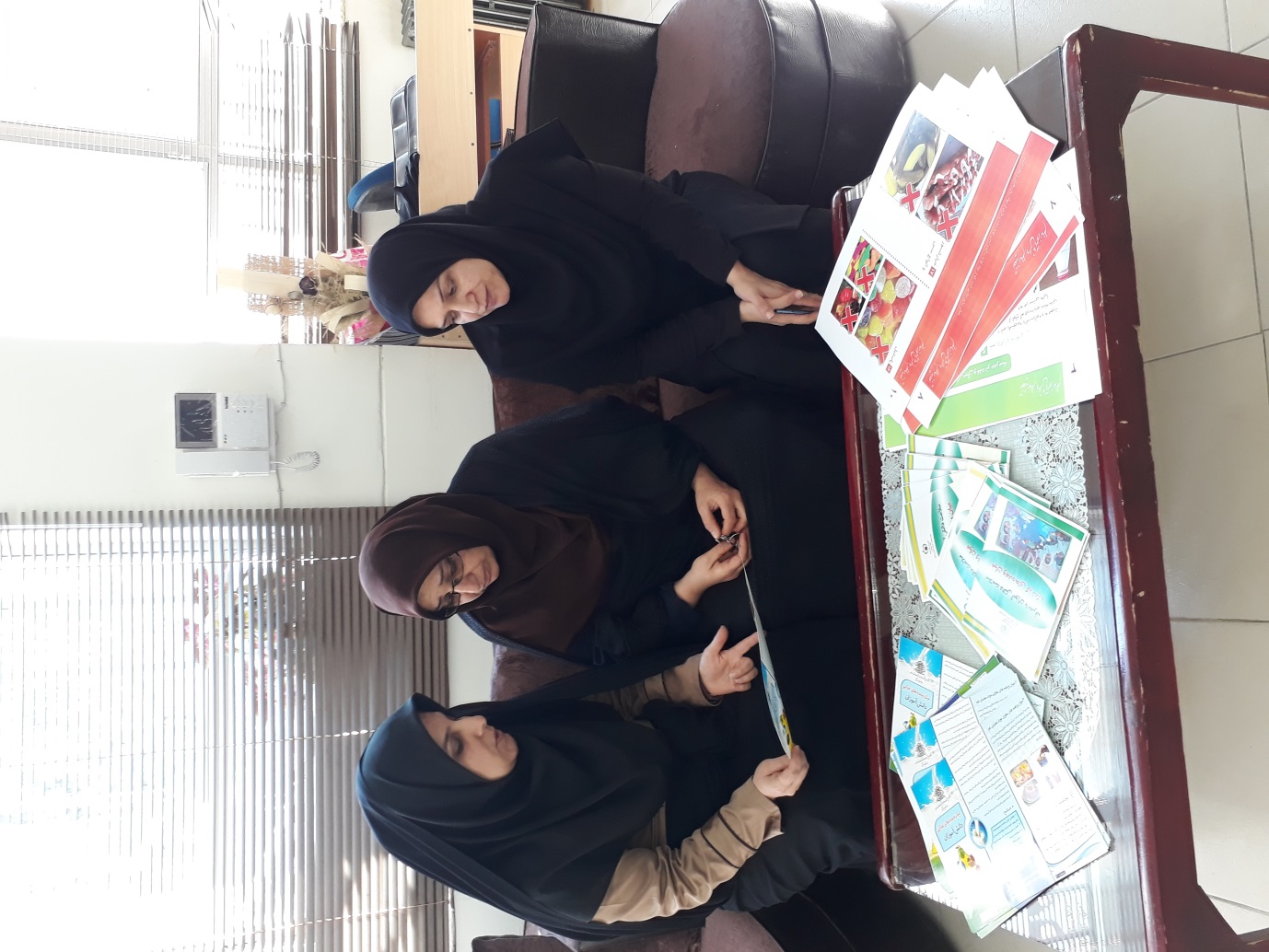


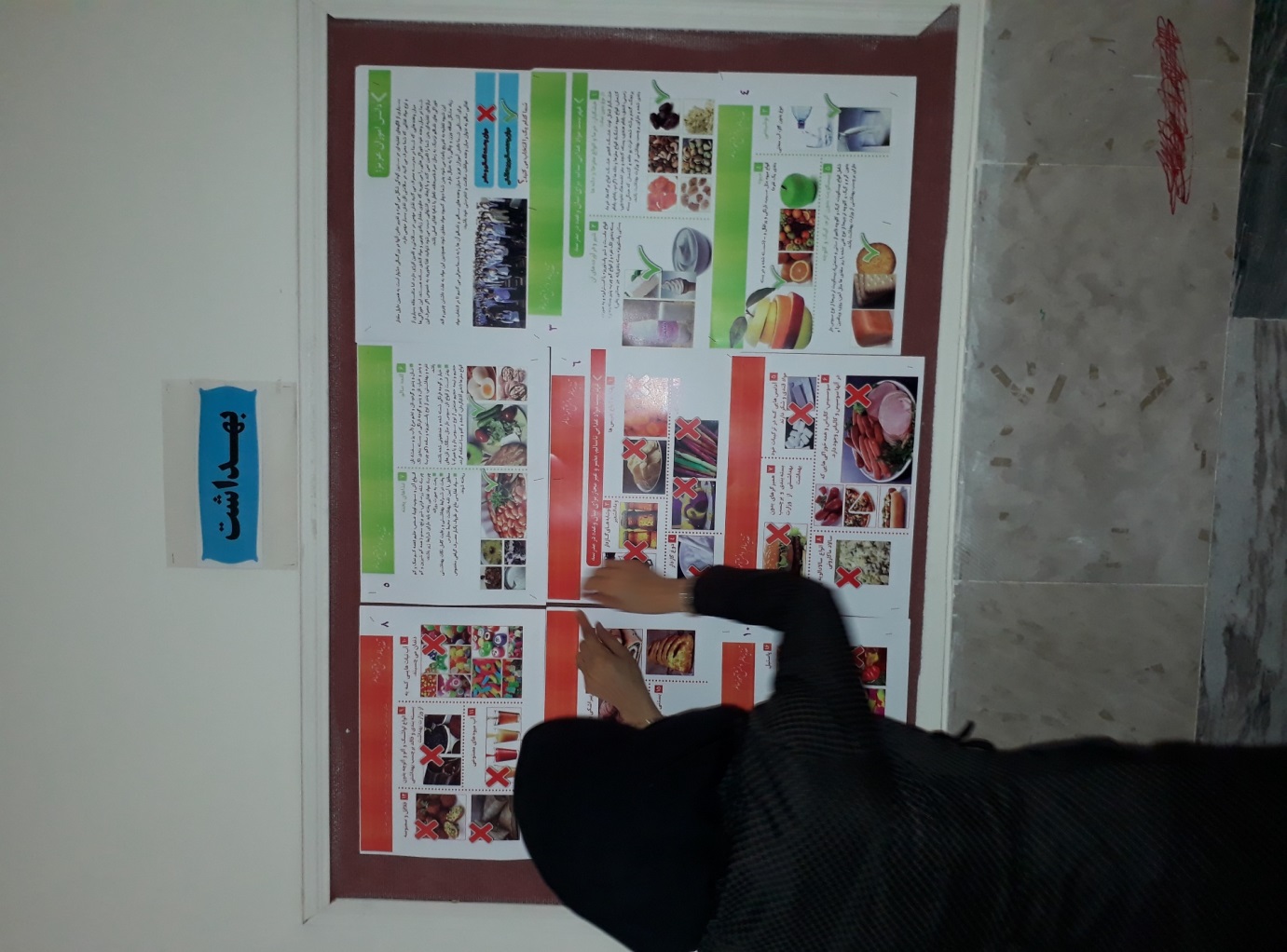


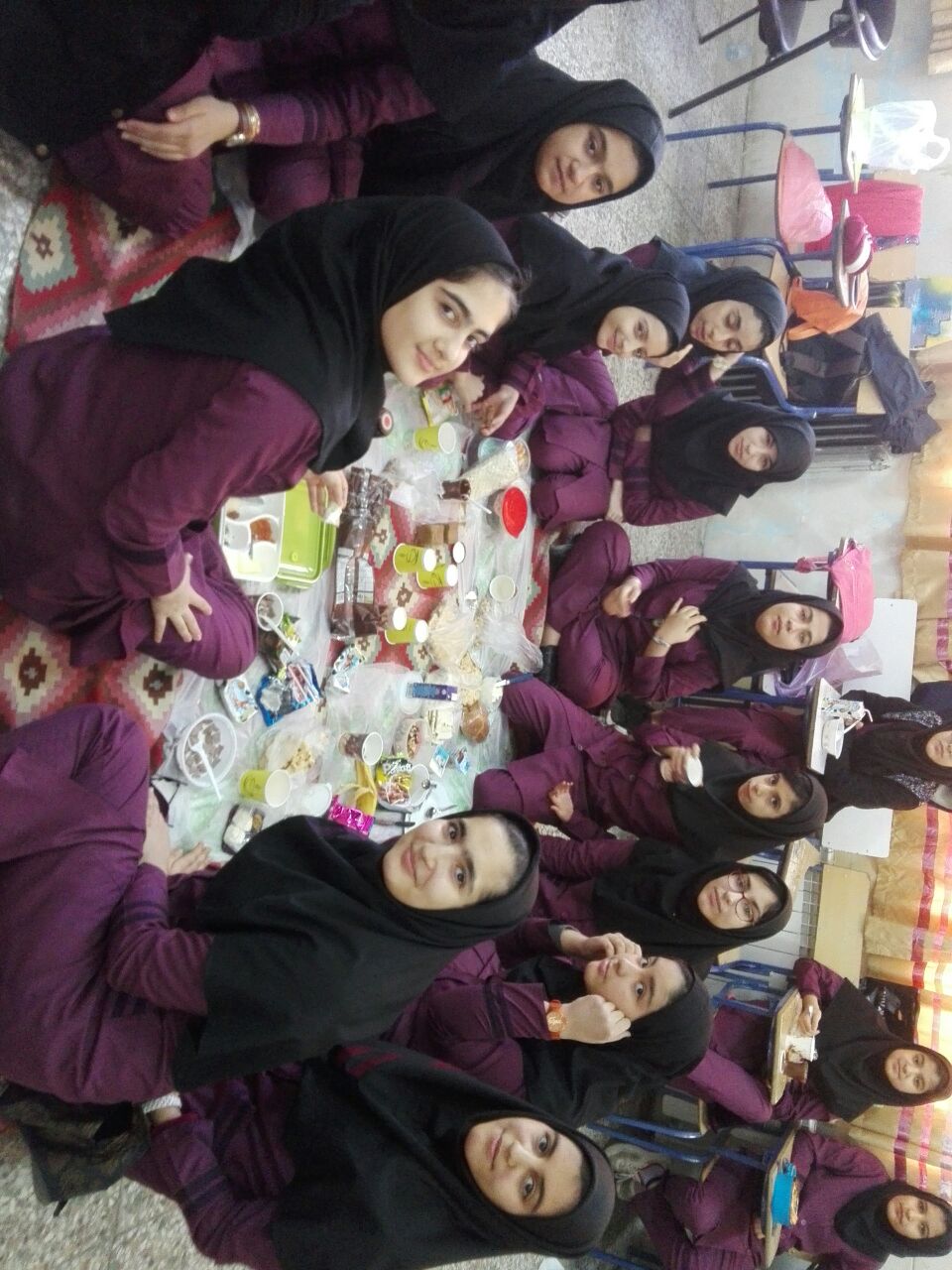


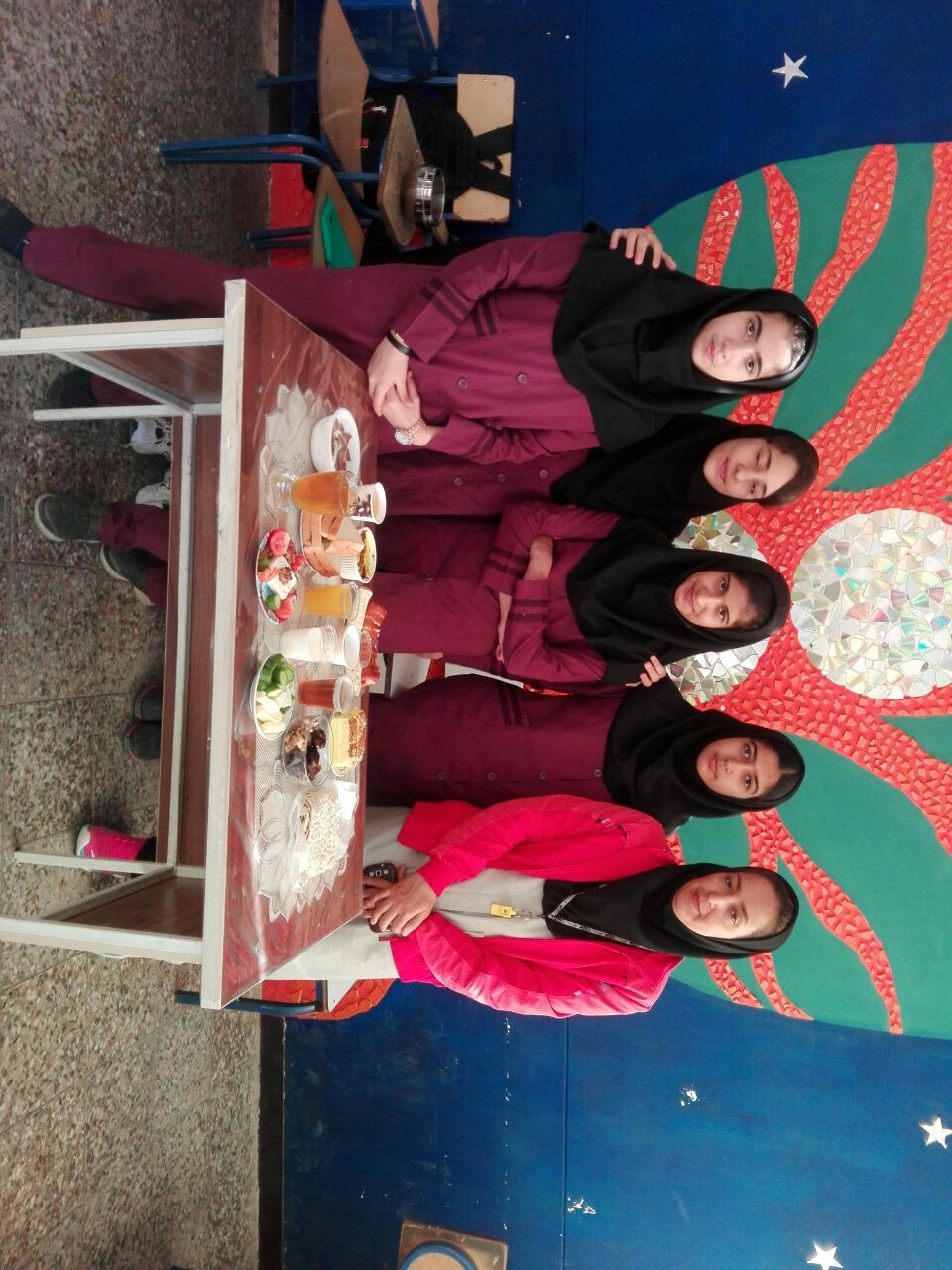


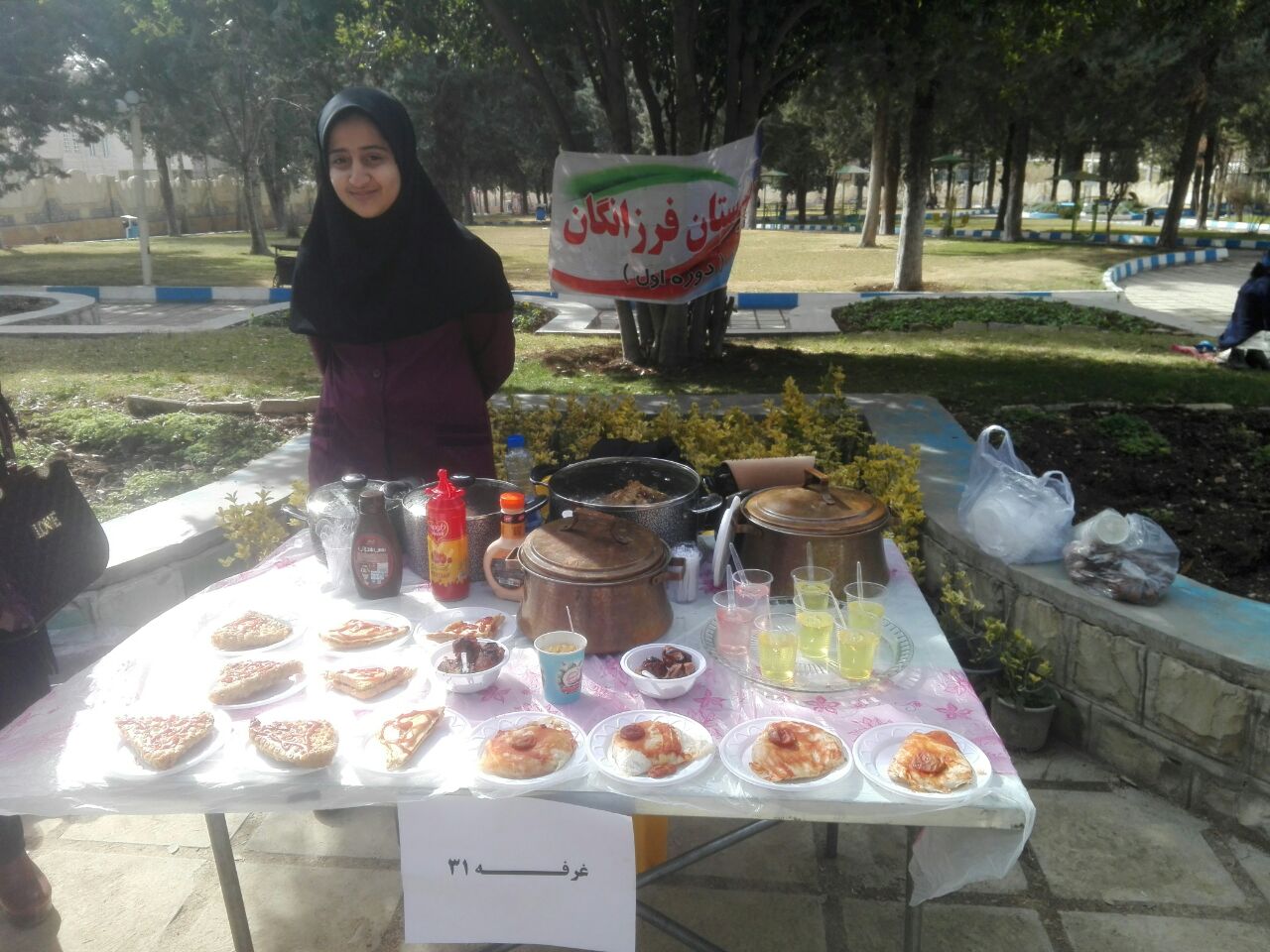

Supplement: Supplementary file 1 — Additional file 1. [file 41043_2021_245_MOESM1_ESM.docx]
